# Supplementary material for: Longitudinal analysis of lipid mediators in post-tuberculosis lung disease identifies significant differences
Source: PLOS Glob Public Health. 2026 Apr 8;6(4):e0006097. doi: 10.1371/journal.pgph.0006097 (PMC13061317; doi:10.1371/journal.pgph.0006097)
Supplement: S1 Appendix — Fig A: CONSORT diagram detailing overall patient recruitment and selection of cases and controls for lipidomic analysis. Table A: Pre-Treatment Cases vs. Controls EBC Lipid Metabolites. Table B: Post-Treatment Cases vs. Post-Treatment Controls EBC Lipid Metabolites. Table C: Linear mixed-effects model to assess longitudinal changes and differences in EBC. Interaction estimates and p values for the ten most significant LM’s by adjusted sequential goodness of fit p-value. S1 Text A: Lipid Glossary. (DOCX) [file pgph.0006097.s001.docx]

APPENDIX:

S1 Fig A: CONSORT diagram detailing overall patient recruitment and selection of cases and controls for lipidomic analysis:


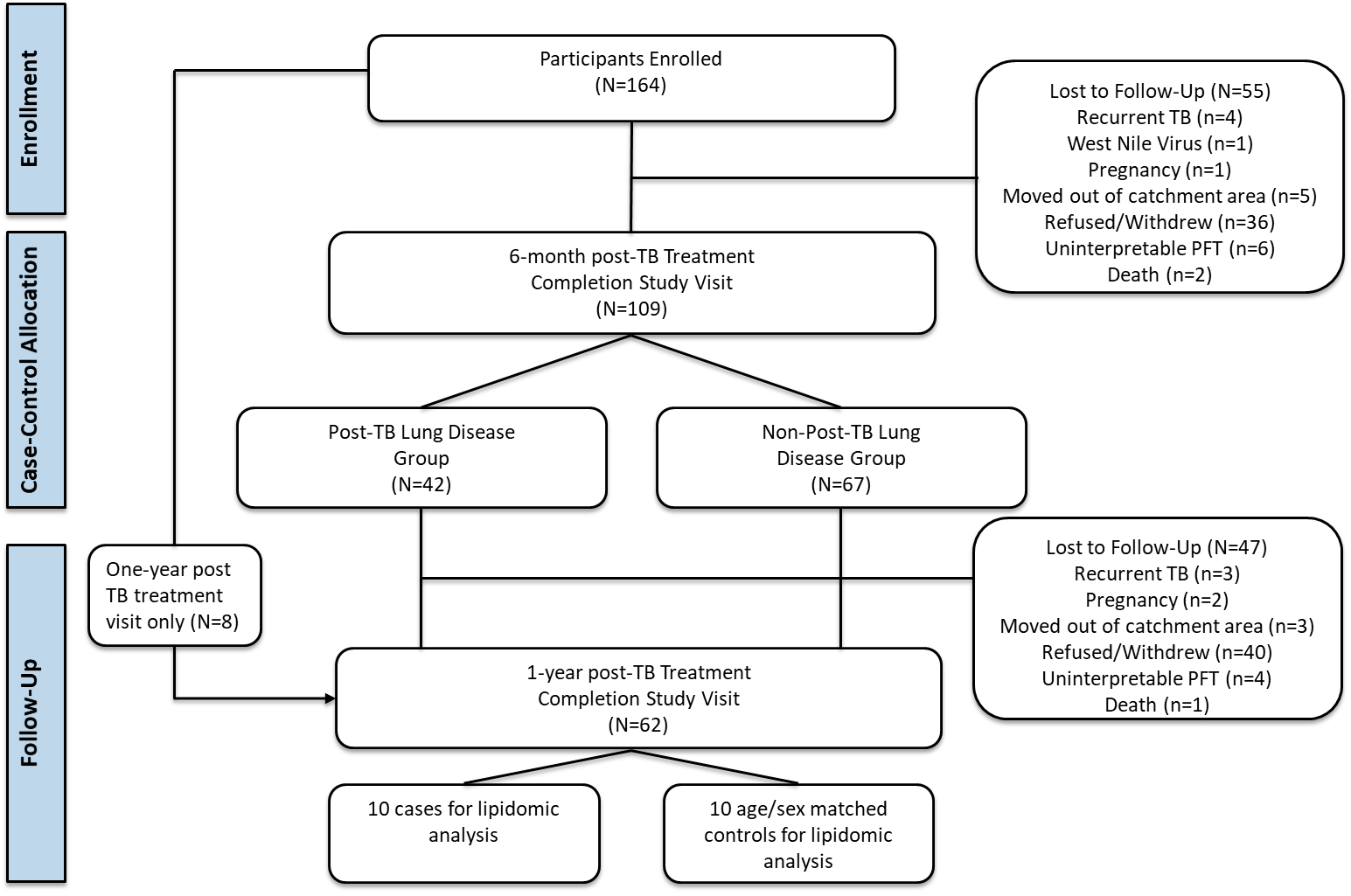


S1 Table A: Pre-Treatment Cases vs. Controls EBC Lipid Metabolites

| Lipid | logFC | AveExpr | t | P.Value | SGoF adj. p |
| --- | --- | --- | --- | --- | --- |
| TXB3 | 0.271 | 0.207 | 2.064 | 0.046 | 0.583 |
| 12(13)-EpOME | 0.132 | 0.102 | 1.463 | 0.152 | 0.659 |
| 11(12)-EpETE | -0.285 | 0.499 | -1.329 | 0.192 | 0.85 |
| 15-oxo LXA4 | -0.332 | 1.377 | -1.219 | 0.23 | 1 |
| 8(S),15(S)-DiHETE | 0.127 | 0.177 | 1.218 | 0.231 | 1 |
| LXA5 | -0.239 | 0.392 | -1.077 | 0.288 | 1 |
| 5(S),15(S)-DiHEPE | 0.129 | 0.195 | 1.019 | 0.314 | 1 |
| 13-14-dh-15k-PGE1 | 0.113 | 0.146 | 0.944 | 0.351 | 1 |
| PGA2 | -0.104 | 0.201 | -0.925 | 0.361 | 1 |

S1 Table B: Post-Treatment Cases vs. Post-Treatment Controls EBC Lipid Metabolites

| Lipid | logFC | AveExpr | t | P.Value | SGoF adj. p |
| --- | --- | --- | --- | --- | --- |
| 14(15)-EpETE | 0.89 | 1.543 | 2.597 | 0.013 | 0.117 |
| 15-oxo LXA4 | 0.627 | 1.377 | 2.306 | 0.027 | 0.142 |
| AT-RvD6 | 0.193 | 0.255 | 2.009 | 0.052 | 0.178 |
| 9-HEPE | 0.144 | 0.07 | 1.943 | 0.059 | 0.18 |
| 15(R)-PGE1 | 0.432 | 0.354 | 1.803 | 0.079 | 0.323 |
| 8-isoPGF2a & 11bPGF2a | 0.557 | 2.357 | 1.682 | 0.101 | 0.346 |
| PGF1a | 0.136 | 0.083 | 1.605 | 0.117 | 0.644 |
| 12(13)-EpOME | 0.14 | 0.102 | 1.553 | 0.129 | 1 |
| PGA2 | 0.169 | 0.201 | 1.498 | 0.142 | 1 |
| 15d-D12-14-PGJ3 | -0.419 | 0.522 | -1.429 | 0.161 | 1 |

S1 Table C: Linear mixed-effects model to assess longitudinal changes and differences in EBC. Interaction estimates and p values for the ten most significant LM’s by adjusted sequential goodness of fit p-value.

| Lipid | Interaction Estimate | Interaction p-value | Interaction SGoF adj. p | Direction of Change |
| --- | --- | --- | --- | --- |
| 18-carboxy dinor LTB4 | 2.16 | 0.068 | 0.756 | Insignificantly Higher |
| 2-hydroxy LTB4 | -0.219 | 0.073 | 0.756 | Insignificantly Lower |
| 15-oxo LXA4 | 0.187 | 0.086 | 0.756 | Insignificantly Higher |
| LXB4 | 1.886 | 0.089 | 0.756 | Insignificantly Higher |
| AT-RvD6 | 0.162 | 0.097 | 0.756 | Insignificantly Higher |
| 12_13-DiHOME | -0.201 | 0.108 | 0.756 | Insignificantly Lower |
| 2-HDoHE | -0.213 | 0.12 | 0.756 | Insignificantly Lower |
| LXA5 | 0.214 | 0.127 | 0.756 | Insignificantly Higher |
| Tetranor PGEM | -0.326 | 0.14 | 0.756 | Insignificantly Lower |
| PD1 | -0.169 | 0.148 | 0.756 | Insignificantly Lower |

**S1 Text A: Lipid Glossary**

10-HDoHE: 10-hydroxy-docosahexaenoic acid

11-12-DiHETrE: 11,12-dihydroxy-eicosatrienoic acid

11-HDoHE: 11-hydroxy-docosahexaenoic acid

11-HETE: 11-hydroxy-eicosatetraenoic acid

12-HEPE: 12-hydroxy-eicosapentaenoic acid

12-HETE: 12-hydroxy-eicosatetraenoic acid

13-HDoHE: 13-hydroxy-docosahexaenoic acid

13(14)-EpDPE: 13(14)-epoxy-docosapentaenoic acid

13-OxoODE: 13-oxo-octadecadienoic acid

14-HDoHE: 14-hydroxy-docosahexaenoic acid

15-HETE: 15-hydroxy-eicosatetraenoic acid

15-oxo LXA4: 15-oxo-lipoxin A4

15-OxoETE: 15-oxo-eicosatetraenoic acid

8-HDoHE: 8-hydroxy-docosahexaenoic acid

8-HEPE: 8-hydroxy-eicosapentaenoic acid

8-HETE: 8-hydroxy-eicosatetraenoic acid

8-HETrE: 8-hydroxy-eicosatrienoic acid

8(9)-EpETrE: 8(9)-epoxy-eicosatrienoic acid

9-HEPE: 9-hydroxy-eicosapentaenoic acid

AT-PD1: Aspirin-triggered protectin D1

AT-RvD6: Aspirin-triggered resolvin D6

iPF-VI: Iso-prostaglandin F-VI

LXB4: Lipoxin B4

MaR2: Maresin2

PD1: Protectin D1

PGA2: Prostaglandin A2
