# Supplementary material for: Longitudinal analysis of lipid mediators in post-tuberculosis lung disease identifies significant differences
Source: PLOS Glob Public Health. 2026 Apr 8;6(4):e0006097. doi: 10.1371/journal.pgph.0006097 (PMC13061317; doi:10.1371/journal.pgph.0006097)
Supplement: Attachment — Submitted filename: Comments, Boadu AA.docx [file pgph.0006097.s002.docx]

Reviewer Comments: PLOS Global Public Health (PGPH-D-25-03241)

Longitudinal Analysis of Lipid Mediators in Post-Tuberculosis Lung Disease Identifies Significant Differences.

**Overview**

This prospective pilot study provides novel hypothesis-generating data on plasma lipid mediator (LM) trajectories predicting post-tuberculosis lung disease (PTLD) in Ugandan TB patients. The LC-MS/MS methodology is rigorous, SGoF correction appropriate for lipidomics, and PD1's paradoxical role elegantly hypothesized. However, methodological inconsistencies, overconfident causal language, and incomplete limitations reporting undermine credibility.

MAJOR REVISIONS

1. Sample Selection & CONSORT Flow (Methods lines 143-153)

Problem: From 109 completers (42 cases/67 controls), n=20 selected based on unquantified high-quality PFTs and one-year follow-up availability (unanalyzed). No power calculation or selection criteria quantified (e.g., % missing data, PFT quality thresholds).

Please add CONSORT flow diagram (Figure S1). Report full cohort baseline characteristics (Table S1). Provide post-hoc power for key LMs (PD1, AT-RvD6). Clarify HIV treatment status (1/10 cases HIV+ despite untreated HIV exclusion).

2. PTLD Definition & Phenotyping (Methods lines 144-146; Table 1)

Problem: Cases = FEV1/FVC Z≤-1.64 OR TLC<80% OR DLCO<80% mixes obstructive/restrictive phenotypes. Table 1: FEV1/FVC Z nonsignificant (p=0.064) despite definition.

Please adopt ATS/ERS PTLD consensus (FEV1<80% predicted + prior TB). Report phenotype breakdown (% obstructive/restrictive/mixed). Sub-stratify LM results if feasible (n=10 limits).

3. Statistical Analysis Clarification (Methods lines 167-182; Table 4)

Problem: Delta-delta = (post-baseline) then log^2^ violates paired assumptions. Table 4 mixes SGoF-significant + insignificant unadjusted p<0.05; cherry-picking.

Please validate with linear mixed models (lme4 R package). Report effect sizes (95%CI). Compare SGoF vs FDR/BH sensitivity. Justify SGoF over standard FDR.

4. EBC Interpretation & Reporting (Results lines 214; 237; 267)

Problem: 92/147 LMs detectable vs 129 plasma; all SGoF p>0.05. Hyped as parallel analysis strength (Discussion line 333) despite null results.

Please report LM recovery rates, dilution correction method, RTube validation. Negative controls?

5. Discussion Overstatement & Structure (Lines 272-351)

Problem: “provides novel evidence for distinct systemic LM profiles... dysregulated resolution pathways in PTLD pathogenesis” 🡪 causal language inappropriate for observational pilot (n=20).

“Replace paragraph 1 opening: “provides novel evidence... dysregulated pathways” 🡪 identifies candidate LM signatures “suggesting” dysregulated resolution warranting validation”

Action: Adopt 3-paragraph structure:

Precise results recap (no repetition)

Literature integration + limitations

Specific hypotheses (e.g., Validate PD1+AT-RvD6 panel AUC in n=200; test 17R-RvD1 1-10μg/kg in PTLD organoids)

**MINOR REVISIONS**

| Section | Issue | Fix |
| --- | --- | --- |
| Abstract line 52 | Fragment: differential... analyses | using differential abundance analyses (limma) |
| Methods line 133 | divided in to 0.5ml | divided into 0.5 mL |
| Methods line 156 | 3 volumes ethanol was added | three volumes of ethanol were added |
| Results line 198 | 10-HDoHE, 11-HDoHE vs Abstract 11-HDoHE | List consistently; confirm isomers |
| Table 3 caption | including including | Remove duplicate |
| Discussion line 280 | EBC analysis we did not | EBC analysis did not |
| Discussion line 306 | LXA4 has been shown to involved | LXA4 has been shown to be involved |
| Author affiliations | KR Maddipati (#5 Miami?) at Wayne State (#4) | Correct to #4 |
| References | Ref 1 (2023 report); Ref 7 missing DOI; Ref 25 ‘Nature Methods 2022 202’ 🡪 Nat Methods 19, 193-204 (2022) | Update to 2025; complete citations or justify |

**TYPO SUMMARY**

ngmL 🡪 ng/mL (Figures 1-3 legends)

sgof.adj.P.Val 🡪 SGoF adj. p (Tables 2-4)

mTB 🡪 *M. tuberculosis* (Discussion line 287)
