# Supplementary material for: Longitudinal analysis of lipid mediators in post-tuberculosis lung disease identifies significant differences
Source: PLOS Glob Public Health. 2026 Apr 8;6(4):e0006097. doi: 10.1371/journal.pgph.0006097 (PMC13061317; doi:10.1371/journal.pgph.0006097)
Supplement: Attachment — Submitted filename: Comments, Boadu AA_PLOS_GH_Addressed.docx [file pgph.0006097.s003.docx]

Reviewer Comments: PLOS Global Public Health (PGPH-D-25-03241)

Longitudinal Analysis of Lipid Mediators in Post-Tuberculosis Lung Disease Identifies Significant Differences.

Reviewer 1:

Jackson and colleagues present a really interesting report on possible mediators of post tuberculosis lung disease. Although the prospective clinical study has a relatively low number of patients, it provides clues for follow-up mechanistic studies on the role of lipid mediators in PTLD.

I think the paper would benefit from the clarification of the following points:

1. Am I correct in assuming that the pulmonary function parameters in Table 1 refer to values collected at visit 2 (post-treatment)? Since the authors refer to it as baseline values, my first impression was that they refer to pre-treatment levels. This should me made clearer for a more general readership.

Thank you for this comment, we have updated the title of this table to reflect that pulmonary function parameters are post-treatment. We did not perform PFT’s during infection due to risk for infection.

2. What was the difference in pulmonary function parameters from visit 1 to visit 2 in cases and controls?

We did not perform pulmonary function testing pre-treatement due to risk of infection. We have added this to line 141-142

3. Is there any information on the lesion extent/type pre-treatment between cases and controls? Could disease severity be linked to the observed patterns?

We agree and are working on other submissions focused on the clinical risk factors for PTLD including radiologic extent. In general this was not consistently related. We believe that this information is beyond the scope and focus of this study specifically looking at LM profiles at various time points for PTLD>

4. Do the authors think that the set of initial conditions (pre-treatment) is the major determinant of PTLD outcomes, or the immune events that occur during treatment are more important? I ask this because, for example, AT-RvD6 is similar pre-treatment but then becomes one of the major differences. Is AT-RvD6 part of the cause or a consequence?

This is difficult to say, as we discuss within the discussion, lines 290-328, there are likely multiple factors. Baseline increases in pro-resolving SPM’s (e.g. PD1) may hinder initial inflammatory response and lower concentrations of pro-resolving SPM’s like AT-RvD6 after treatment may suggest that there is dysregulated resolution with suppressed inflammation during acute infection and too little resolving mediators when the microbiological infection has been treated. Further work to understand all of the factors relating to LM modulation of immunologic response at different time points is certainly necessary.

**Reviewer 2:**

**Overview**

This prospective pilot study provides novel hypothesis-generating data on plasma lipid mediator (LM) trajectories predicting post-tuberculosis lung disease (PTLD) in Ugandan TB patients. The LC-MS/MS methodology is rigorous, SGoF correction appropriate for lipidomics, and PD1's paradoxical role elegantly hypothesized. However, methodological inconsistencies, overconfident causal language, and incomplete limitations reporting undermine credibility.

MAJOR REVISIONS

1. Sample Selection & CONSORT Flow (Methods lines 143-153)

Problem: From 109 completers (42 cases/67 controls), n=20 selected based on unquantified high-quality PFTs and one-year follow-up availability (unanalyzed). No power calculation or selection criteria quantified (e.g., % missing data, PFT quality thresholds).

Please add CONSORT flow diagram (Figure S1). Report full cohort baseline characteristics (Table S1). Provide post-hoc power for key LMs (PD1, AT-RvD6). Clarify HIV treatment status (1/10 cases HIV+ despite untreated HIV exclusion).

We have added a CONSORT diagram to show selection for our overall study and the selection of our 10 cases and controls supplementary figure 1. While we recognize the interest our overall cohort demographics has this is being submitted actively as another study, this study is a nested study within our larger descriptive longitudinal cohort of PTLD. We wish to be congizant of the amount of figures and data presented and do not believe it adds significant interest to the paper. Our population who did have follow up is copied here for your review:

| **Characteristics** | **Overall**  (N= 109) | **PTLD**  (n=42) | **Non-PTLD**  (n=67) | **p-value** |
| --- | --- | --- | --- | --- |
| **Age, years, mean (SD)** | 34.0 (11.0) | 34.8 (12.4) | 33.2 (9.8) | 0.806 |
| **Male sex, n (%)** | 78 (71.6%) | 29 (69.0%) | 49 (73.1%) | 0.809 |
| **BMI kg/m^3^, mean (SD)** | 19.8 (3.2) | 19.2 (2.8) | 20.3 (3.4) | 0.079 |
| **Ever Smoker, n (%)** | 24 (22.0%) | 11 (26.2%) | 13 (19.4%) | 1.00 |
| **Food Insecurity, mean (SD)** | 3.5 (5.1) | 3.9 (5.1) | 3.7 (4.4) | 0.872 |
| **Total WAMI Score, mean (SD)** | 0.5 (0.1) | 0.5 (0.1) | 0.5 (0.2) | 0.159 |
| **Household Income (USD), mean (SD)** | 1491.3 (3744.5) | 1184.3 (2005.4) | 1683.7 (4510.9) | 0.500 |
| **Self-Reported HIV Diagnosis, n (%)** | 15 (13.8%) | 5 (11.9%) | 10 (14.9%) | 1.00 |
| **Self-Reported DM Diagnosis, n (%)** | 3 (2.8%) | 1 (2.4%) | 2 (3.0%) | 0.851 |
| **Responsible for household biomass cooking, n (%)** | 54 (49.5%) | 21 (50.0%) | 33 (49.3%) | 1.000 |
| **Daily Biomass Cooking, n (%)** | 69 (63.3%) | 29 (69.0%) | 40 (59.7%) | 0.821 |

Post hoc (achieved) power was calculated for the requested plasma lipids using the limma model t-statistics by converting each t value into an implied standardized effect size (Cohen’s d) under a two-group comparison with equal group sizes, and then computing two-sided power at alpha 0.05 using the noncentral t distribution. Degrees of freedom were inferred by matching the reported two-sided p-values to the Student t distribution, yielding df = 37 for these plasma contrasts, corresponding to an effective n ≈ 19.5 per group. At baseline, achieved power ranged from 0.084 (12-HETE) to 0.888 (PD1), with intermediate values for PGA2 0.779, 13(14)-EpDPE 0.763, 11-HDoHE 0.643, and 12-HEPE 0.454. Post-treatment, achieved power was very high for both AT-RvD6 (1.000; mapped to the AT-RvD6-related feature at_rvd6_4_17_dihdohe in the dataset) and 15-oxo-LXA4 (0.981). This was no added to the text but we are happy to add this should the reviewers feel it is necessary.

We have clarified that the one subject in the PTLD group had HIV with an undetectable viral load per chart review in line 153.

2. PTLD Definition & Phenotyping (Methods lines 144-146; Table 1)

Problem: Cases = FEV1/FVC Z≤-1.64 OR TLC<80% OR DLCO<80% mixes obstructive/restrictive phenotypes. Table 1: FEV1/FVC Z nonsignificant (p=0.064) despite definition.

Please adopt ATS/ERS PTLD consensus (FEV1<80% predicted + prior TB). Report phenotype breakdown (% obstructive/restrictive/mixed). Sub-stratify LM results if feasible (n=10 limits).

We feel we have adequately discussed this choice to include subjects with abnormal lung function as our inclusion for PTLD. However, we have reviewed our samples and all controls had FEV1 >80% min 82% and max 113%, SD 7%, Mean 97% and our cases all had FEV1 <80% predicted, min 45%, max 77%, SD 9%, Mean 64%. Please cite the reference for FEV1 <80% and prior TB, the recent post-TB lung conference in South Africa has acknowledged that PFT abnormalities of all types are an important aspect of PTLD diagnosis and do not make mention of this definition. <https://doi.org/10.1016/S1473-3099(25)00548-1>. We have added the phenotype of PFT changes in lines 193-196.

Substratification of LM results was not possible due to small sample size.

3. Statistical Analysis Clarification (Methods lines 167-182; Table 4)

Problem: Delta-delta = (post-baseline) then log^2^ violates paired assumptions. Table 4 mixes SGoF-significant + insignificant unadjusted p<0.05; cherry-picking.

Please validate with linear mixed models (lme4 R package). Report effect sizes (95%CI). Compare SGoF vs FDR/BH sensitivity. Justify SGoF over standard FDR.

We have completed this analysis with linear mixed models and have included these in the paper. We have updated table with the smallest SGoF p values for this analysis and have updated the figure with the 6 most significant lipids by SgoF p values as bar charts to demonstrate changes overtime. There was only one LM PD1 that was <0.05 by SgoF p value and we have updated our discussion and results portion to reflect this and changed the tone of the language to describe the other LM’s lines 251-267

Regarding the FDR and BH sensitivity vs. SGoF we feel we adequately justified our decision to use SGoF based upon citation from experts in the field of lipidomic analysis suggesting this methodology (Ni Z, Wölk M, Jukes G, et al. Guiding the choice of informatics software and tools for lipidomics research applications. Nature Methods 2022 20:2. 2022;20(2):193-204. doi:10.1038/s41592-022-01710-0).

To respond fully to this comment we have prepared the following figures for plasma and EBC to compare SgoF vs. BH and present them here.

Demonstrates that SGoF P-values for Plasma are more flexible at low alpha values but then becomes very conservative within moderate alpha values
On the other hand, Benjamini-Hochberg (bh) is very conservative from low to moderate and high alpha values

Demonstrates that SGoF P-values for EBC are more flexible at low alpha values but then becomes very conservative within moderate alpha values
On the other hand, Benjamini-Hochberg (bh) is very conservative from low to moderate and high alpha values

4. EBC Interpretation & Reporting (Results lines 214; 237; 267)

Problem: 92/147 LMs detectable vs 129 plasma; all SGoF p>0.05. Hyped as parallel analysis strength (Discussion line 333) despite null results.

Please report LM recovery rates, dilution correction method, RTube validation. Negative controls?

We have expanded our discussion regarding EBC results in discussion lines 328-338. The LM recovery rates show that there were 84.4% of lipids at the lower limit of detection compared to 49.7% in plasma. We also report quality analysis to show internal standards and quality of our EBC analysis. There was no dilution correction performed given lack of gold standard marker for EBC, pilot nature of the study and the risk of bias with correction (this was added in line 159-161). Our methodology for R-tube usage is well justified, we utilized standards for R-tube analysis and cite previous studies using this platform by our group where differences in analytes existed for other lung diseases e.g. COPD. (lines 132-135 and 345-347).

We did not use negative r-tube blanks within this study, this is not necessarily standard given that Wayne state using reagent blanks to check for contamination and that we found a high percentage of LM’s at the lower limit of detection suggesting that there were not significant LM’s contaminating samples.

5. Discussion Overstatement & Structure (Lines 272-351)

Problem: “provides novel evidence for distinct systemic LM profiles... dysregulated resolution pathways in PTLD pathogenesis” 🡪 causal language inappropriate for observational pilot (n=20).

We have edited this portion to temper our findings in line 279.

“Replace paragraph 1 opening: “provides novel evidence... dysregulated pathways” 🡪 identifies candidate LM signatures “suggesting” dysregulated resolution warranting validation”

Action: Adopt 3-paragraph structure:

Precise results recap (no repetition)

Literature integration + limitations

Specific hypotheses (e.g., Validate PD1+AT-RvD6 panel AUC in n=200; test 17R-RvD1 1-10μg/kg in PTLD organoids)

We have adjusted the opening statement of our discussion and have modified line 363 to alude to larger cohorts to validate specific LM’s. We appreciate this comment but have left some of the language given that our group is determining the best path forward within our research and while we appreciate the need for specific hypothesis in grant proposals we are reticent to state specific hypothesis and methadology for future work prior to determining the best way forward.

**MINOR REVISIONS**

| Section | Issue | Fix |
| --- | --- | --- |
| Abstract line 52 | Fragment: differential... analyses | using differential abundance analyses (limma) |
| Methods line 133 | divided in to 0.5ml | divided into 0.5 mL |
| Methods line 156 | 3 volumes ethanol was added | three volumes of ethanol were added |
| Results line 198 | 10-HDoHE, 11-HDoHE vs Abstract 11-HDoHE | List consistently; confirm isomers |
| Table 3 caption | including including | Remove duplicate |
| Discussion line 280 | EBC analysis we did not | EBC analysis did not |
| Discussion line 306 | LXA4 has been shown to involved | LXA4 has been shown to be involved |
| Author affiliations | KR Maddipati (#5 Miami?) at Wayne State (#4) | Correct to #4 |
| References | Ref 1 (2023 report); Ref 7 missing DOI; Ref 25 ‘Nature Methods 2022 202’ 🡪 Nat Methods 19, 193-204 (2022) | Update to 2025; complete citations or justify |

We have corrected these minor revisions. Ref 25 citation exported to mendelay maintains this formatting despite exporting the citation and reviewing the citation. We have elected to report the citation as exported.

**TYPO SUMMARY**

ngmL 🡪 ng/mL (Figures 1-3 legends) – corrected

sgof.adj.P.Val 🡪 SGoF adj. p (Tables 2-4) - corrected

mTB 🡪 *M. tuberculosis* (Discussion line 287) - corrected
